# Metabolic syndrome and risk of stroke: Protocol for an update systematic review and meta-analysis

**DOI:** 10.1097/MD.0000000000009862

**Published:** 2018-04-13

**Authors:** Leonardo Roever, Elmiro Santos Resende, Angélica Lemos Debs Diniz, Nilson Penha-Silva, João Lucas O’Connell, Paulo Fernando Silva Gomes, Hugo Ribeiro Zanetti, Anaisa Silva Roerver-Borges, Fernando César Veloso, Thiago Montes Fidale, Antonio Casella-Filho, Paulo Magno Martins Dourado, Antonio Carlos Palandri Chagas, Sadeq Ali-Hasan-Al-Saegh, Paulo Eduardo Ocke Reis, Rogério de Melo Pinto, Gustavo B.F. Oliveira, Álvaro Avezum, Mansueto Neto, André Durães, Rose Mary Ferreira Lisboa da Silva, Antonio José Grande, Celise Denardi, Renato Delascio Lopes, Nitesh Nerlekar, Shahab Alizadeh, Adrian V. Hernandez, Giuseppe Biondi-Zoccai

**Affiliations:** a Federal University of Uberlândia, Department of Clinical Research; b Heart Institute (InCor), Master Institute of Education President Antonio Carlos, Department of Clinical Research, IMEPAC, Araguari, Brazil; c HCFMUSP—University of São Paulo Medical School, Department of Cardiology, São Paulo; d Faculty of Medicine ABC, Department of Cardiology Santo André, Brazil; e Cardiovascular Research Center, Shahid Sadoughi University of Medical Sciences, Department of Cardiology, Yazd, Iran; f Department of Specialized and General Surgery, Fluminense Federal University, Rio de Janeiro; g Dante Pazzanese Institute of Cardiology, São Paulo, Brazil; Dante Pazzanese Institute of Cardiology, Department of Clinical Research São Paulo; h Graduate Program in Medicine and Health, Department of Heath and Sciences, Federal University of Bahia; i Federal University of Minas Gerais, Department of Cardiology, MG; j Federal University of Mato Grosso, MT, Department of Medicine, Brazil; k FOP Unicamp, Department of Clinical Research; l Division of Cardiology, Duke University Medical Center, Department of Clinical Research, Durham, NC; m Monash Cardiovascular Research Centre and Monash Heart, Department of Cardiology, Clayton, Victoria, Australia; n Tehran University of Medical Sciences, Department of Medicine, Iran; o University of Connecticut/Hartford Hospital Evidence-Based Practice Center, Hartford, Department of Comparative Effectiveness and Outcomes Research Health Outcomes, CT; p Department of Medico-Surgical Sciences and Biotechnologies, Sapienza University of Rome, Latina, Italy Department of Angiocardioneurology, IRCCS Neuromed, Pozzilli, Italy.

**Keywords:** metabolic syndrome, stroke, systematic review

## Abstract

**Introduction:**

The metabolic syndrome is composed of several cardiovascular risk factors and has a high prevalence throughout the world. However, there are no systematic analyses or well-conducted meta-analyses to evaluate the relationship between metabolic syndrome and stroke. The aim of this study is to examine this association of metabolic syndrome with stroke in different ages and sex.

**Methods and analysis:**

The update systematic review and meta-analysis will be conducted using published studies that will be identified from electronic databases (i.e., PubMed, EMBASE, Web of Science, and Google Scholar. Studies that examined the association between metabolic syndrome and stroke, had a longitudinal or prospective cohort design, were conducted among in adults aged 40 to 70 years, provided sufficient data for calculating ORs or relative risk with a 95% CI, were published as original articles written in English or other languages, and have been published until December 2017 will be included. Study selection, data collection, quality assessment, and statistical syntheses will be conducted based on discussions among investigators.

**Ethics and dissemination:**

Ethics approval was not required for this study because it was based on published studies. The results and findings of this study will be submitted and published in a scientific peer-reviewed journal. The findings from this study could be useful for assessing metabolic syndrome risk factors in stroke, and determining approaches for prevention of stroke in the future.

## Abstract 2

Supplemental Digital Content is available in the text

## 1 Strengths and limitations of this study

This systematic review and meta-analysis will offer better understanding regarding the association between metabolic syndrome and stroke. The findings from this study will be useful for assessing composite metabolic syndrome risk factors in stroke, and determining approaches for prevention of stroke the future. An improved understanding of this relationship may help to inform public health stroke prevention strategies.

Included studies may have substantially different methodologies, which could limit our ability to draw reliable conclusions from the existing evidence base. Depending on the results, confounding factors that were not adjusted for in the selected studies and low generalizability can be limitations. Individual patient data will not be available.

## 2 Background

The prevalence of metabolic syndrome (MetS) and MetS-related stroke is set to increase dramatically in coming decades. MetS is a complex disease that includes endothelial dysfunction, insulin resistance, diabetes, hypertension, ectopic obesity, and dyslipidaemia and an increased risk of cardiovascular events. It is in large part the result of unbalanced diet, low socioeconomic and cultural level, stress and sedentary lifestyle. Although the literature on the MetS and the risk factors for stroke has been increasing, to our knowledge, a systematic review of the association between MetS and risk of stroke has not yet been conducted.^[1–12]^ This study aims to systematically assess the association between MetS and stroke in adults aged 40 to 70 years; and to provide a framework to further understand these factors in order to better target prevention strategies.

## 3 Methods/design

This systematic review of the literature will follow the Preferred Reporting Items for Systematic Reviews and Meta-Analyses (PRISMA) recommendations. The databases PubMed, Embase, Web of Science, Google Scholar, and Cochrane were searched for articles. Our search will focus on cohort, case–control and cross-sectional studies examining the association between MetS and stroke. The primary outcome is stroke. Two reviewers will independently screen articles, extract relevant data and assess the quality of the studies.

MetS will be defined according to the unified definition of metabolic syndrome based on a Joint Interim Statement of the International Diabetes Federation Task Force on Epidemiology and Prevention; National Heart, Lung, and Blood Institute; American Heart Association; World Heart Federation; International Atherosclerosis Society; and International Association for the Study of Obesity. We also consider the patients with the MetS diagnosis based on previous definitions, mainly based on the National Cholesterol Education Program's Adult Treatment Panel III. Individuals will be classified as having MetS if they have 3 or more of the followings from UHS Visit 1: elevated BP (systolic BP ≥130 mm Hg or diastolic BP ≥85 mm Hg); elevated TG (≥150 mg/dL); low HDL-C (men <40 mg/dL, women <50 mg/dL); impaired fasting glucose (>100 mg/dL); and elevated waist circumference (WC) (men ≥94 cm, women ≥80 cm). Diabetes will be defined as a fasting plasma glucose level ≥126 mg/dL. Impaired fasting glucose will be defined as a fasting plasma glucose level of 100 to 125 mg/dL among those not treated for diabetes.^[1,2]^ The study is registered with PROSPERO (CRD42016049917). This protocol conforms to the Preferred Reporting Items for Systematic Reviews and Meta-Analyses Protocols (PRISMA-P) guidelines.^[13,14]^

## 4 Systematic review registration

This protocol is registered in the PROSPERO registry of the University of York (Reference number: CRD42016049917).

## 5 Objectives

The primary objective is to identify and summarize the associated with MetS diagnosis and with stroke risk in adults (34–70 years) in different ages and sex.

## 6 Eligibility criteria

The PICOS strategy (population, intervention [changed to exposure for the purposes of this review of observational studies], comparator, outcome, study characteristics) was used to define the eligibility criteria for this study:

Data items on the following 5 domains will be extracted:

1. *Population:* characteristics of the study population (eg, mean/median age, ethnic distribution), inclusion and exclusion criteria.
2. *Exposure:* definition and identification of MetS.
3. *Comparators:* definition and identification of unexposed individuals, number of unexposed subjects.
4. *Outcomes:* definition and identification of primary (stroke) and secondary outcomes (stroke subtypes or TIA), number of subjects with outcome.
5. *Study characteristics:* authors, publication year, setting/source of participants, design, methods of recruitment and sampling, period of study, length of follow-up time (if relevant), aims and objectives.

## 7 Study design

**Figure 1:**
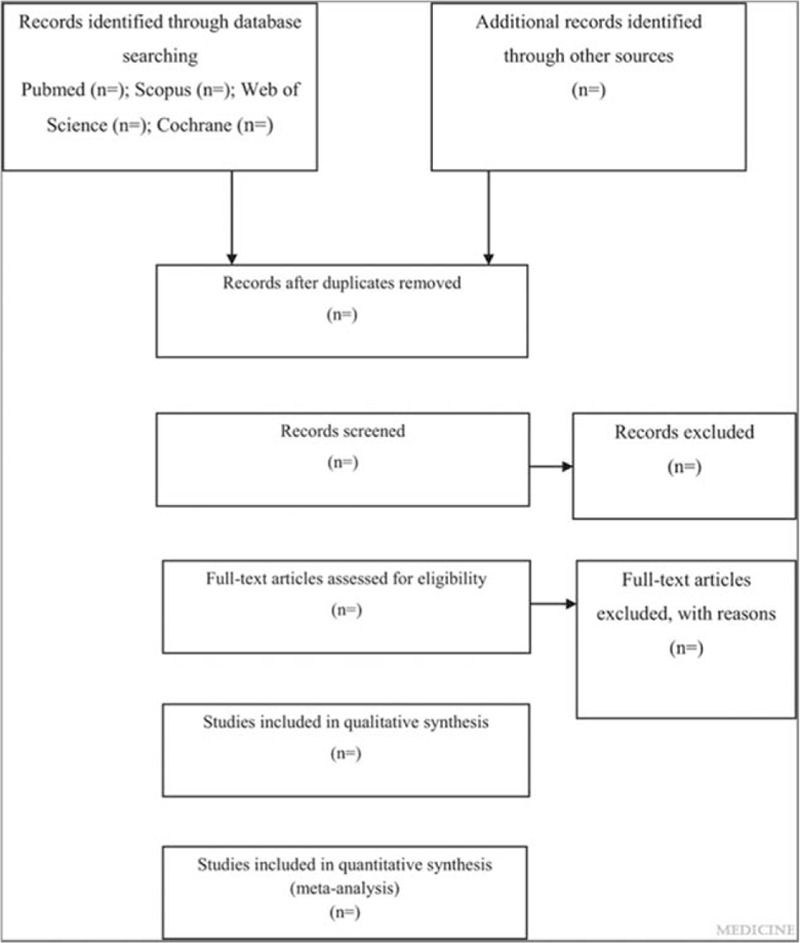
Flow diagram of study selection process.

This is a systematic review and meta-analysis protocol of prospective cohort studies, following the PRISMA-P (Preferred Reporting Items for Systematic Reviews and Meta-Analysis protocols) guideline.^[14]^ The systematic review and meta-analysis will be reported according to the PRISMA (Preferred Reporting Items for Systematic Reviews and Meta-Analyses) guideline.^[15]^ The whole process of study selection is summarized in the PRISMA flow diagram (Fig. 1).

The study protocol was registered at PROSPERO (CRD42016049917).

## 8 Search strategy

A systematic review of the literature will be conducted. A language restriction shall not be applied to the search. If there are relevant non-English abstracts, attempts shall be made to translate them wherever possible. The following bibliographic databases (Embase, PubMed-MEDLINE, Web of Science, Cochrane Library, and Google Scholar) will be searched for articles published until January 2018.

In regard to gray literature, the proceedings of the *International Stroke Conference, European Stroke Conference, European Stroke Organization, World Stroke Organization*, and the *Annual General Meeting of the American Heart Association* will be searched. Several approaches will be undertaken to increase our retrieval of relevant articles. The journals *Stroke*, *International Journal of Stroke, Journal of Stroke and Cerebrovascular Diseases, Lancet Neurology, Diabetes, Neuroradiology, and American Journal of Neuroradiology* will be hand searched to ensure studies have not been missed. These journals are considered to be of the highest impact for the clinical subject of interest.

The literature search strategy is included in Additional file S1. Our search focuses on studies examining the association between MetS diagnosis and stroke risk in adults (34–70 years).^[11]^ At each step of the selection process, reasons for inclusion/exclusion will be recorded in the PRISMA Flowchart.^[13]^

## 9 Data collection

A record will be kept of all searches and search decisions to ensure reproducibility. Search results will be exported to a citation management program (EndNote ver. 7.0). Duplicates will be removed and retained separately. The resulting references will be exported separately to the 2 reviewers for independent review using MS Excel.

## 10 Selection of studies

Two authors (LR and FCV) will independently screen all titles and abstracts identified through the literature searches and will exclude all records clearly not meeting inclusion criteria. Disagreements will be resolved by consensus. The selection process will be pilot tested to ensure a high degree of agreement between reviewers. Full text of the remaining studies will then be retrieved. The same 2 authors (LR and FCV) will independently assess the papers for fulfilment of inclusion criteria. In case of differences of opinion regarding study inclusion, a third review author (GBZ) will serve as arbiter. To avoid double counting, if multiple publications based on the same cohort of participants are retrieved, only the study reporting the largest sample size will be used. The reasons for excluding papers for which the full text was retrieved will be documented.

## 11 Data extraction and management

A data extraction form will be used to collect details from the included studies. The form includes information on study design, patient population, and presence of stroke. Two review authors (LR and FCV) will independently extract the data. The data extraction form will be pilot tested on several papers to ensure consistency and that all relevant information is being captured. If necessary, a statistician will review the extraction of data to further ensure quality and reliability. Authors will be contacted for missing data.

Data will be extracted using a standardized template. We will use the PICOS (Population, Intervention, Comparator, Outcomes and Study design) framework, originally devised to formulate a research question, as a basis to develop data extraction criteria. As this is an aetiological study, “exposure” will replace “intervention” and “study characteristics” will replace “study design.”

In terms of the study results, unadjusted and fully adjusted effect estimates for the association between MetS and stroke will be recorded. Details of the confounders measured and adjusted for will also be noted. Results of any additional stratified analyses will also be recorded. Where possible, results from additional subgroup analyses with evidence regarding our nonprimary objectives will also be recorded, for example, the association between MetS and the secondary outcomes (stroke subtype or TIA).

Bibliographic software (Endnote) will be used for the data management of retrieved references. All the results of the literature searches will be imported into the program and duplicates removed by the main reviewer (LR).

## 12 Outcomes

### 12.1 Primary outcomes

All Stroke (relative risk [RR] and odds ratio [OR]). Studies will be included in the review if the primary outcome was any stroke, clinically diagnosed or self-reported, and the patient's first ever or subsequent stroke.

For studies meeting the inclusion criteria, we will additionally assess the following secondary outcomes: TIA (a transient episode of neurological dysfunction caused by focal brain, spinal cord or retinal ischaemia without acute infarction) and subtypes of stroke (ischaemic vs hemorrhagic). Most strokes (approximately 85%) are ischaemic (an episode of neurological dysfunction caused by focal, cerebral, spinal, or retinal infarction), compared with hemorrhagic (neurological dysfunction caused by a focal collection of blood within or on the surface of the brain). Eligibility criteria may be further developed, in an iterative process, after preliminary searches.

## 13 Assessment of methodological quality

Two investigators (LR and FCV) will independently assess each selected study for study quality using the Newcastle–Ottawa Quality Assessment Scale (NOS).^[16]^ The NOS evaluates cohort studies based on 8 items categorized into the following 3 groups: selection of the study cases, comparability of the population, and ascertainment of whether the exposure or outcome includes any risk of bias (i.e., selection bias or bias from lost to follow-up). The NOS is scored ranging from 0 to 9, and studies with scores ≥7 are considered as high quality.^[16]^ Discrepancy of quality assessment among the investigators will be solved by discussion and consensus among all authors.

## 14 Data synthesis and statistical analysis

We anticipate that there may be significant heterogeneity in the prevalence of MetS features of stroke. There are several factors that could contribute to such heterogeneity. The relative risk (RR) and odds ratio (OR) are the way the result will be expressed statistically.

These factors include the following: differences in demographic and clinical features (e.g., age, hypertension, renal disease, smoking, duration, and severity of diabetes) among study cohorts; differences in definitions of MetS. An *I*^2^ statistic will be calculated for the studies to be included in each proposed meta-analysis (i.e., for each neuroradiology correlate of interest) with values of 25%, 50%, and 75% suggesting low, moderate, or high degrees of heterogeneity, respectively, which report a dichotomized (i.e., present or absent) or categorical (i.e., absent, mild, moderate, severe) shall be harmonized for meta-analysis if deemed appropriate by our statistician. Other types of rating scales shall not be included in a meta-analysis and the data based on any such data scale would be presented in narrative form.

If significant heterogeneity between studies, as determined by consultation with our statistician, prevents meaningful pooling of the data, we will limit ourselves to providing a narrative description of observed trends. Given the heterogeneity of the populations studied, assumption of a fixed effect size across populations would not be justified, thus analyses would be performed using a random effects model. Given the dichotomized (presence or absence) or categorical (severity measure) nature of our data of, meta-analysis will be performed a random effects analysis. We will also add funnel graphs, publication bias analysis and a meta-regression analysis that were not included in previous meta-analyses.^[17,18]^

If there are sufficient data to allow such analyses (in principle from as few as a single high quality study, but if possible by pooling data from multiple studies), we will perform subgroup analyses for participants with renal disease and participants with hypertension. In addition, if sufficient data are available, we shall perform subgroup analyses by age and diabetes duration. Funding sources and conflict of interest will be extracted from included studies. Statistical analysis will be performed using RevMan software.

## 15 Summary of evidence

We will produce a narrative synthesis of the main results extracted from articles in full text. A summary of the included studies will provide information on the authors, study design, participants, number and age of the subjects, theoretical structure (if relevant), alcohol consumption (as primary outcome of interest), main findings, Study information. Special emphasis will be placed on the identification of MetS and the risk of stroke. In the presentation of the results, we will try to separate the factors for which the evidence of causality is strong (from longitudinal studies) and factors for which the causal nature of the relationship is less secure (cross-sectional data). A graphical summary of all the data they represent will be provided and take into account the number of studies that provide evidence of a factor and the relative strength of the association presented based on study design and quality assessment. The membership level will be evaluated based on adjusted data.

## 16 Discussion

This systematic review will synthesize research evidence to establish whether the risk of developing stroke is relatively high in adults with MetS. Strengths and limitations will be highlighted in the identified evidence. Strength of observational data may include large sample size, high rate of follow-up and frequency of stroke more likely to be representative of the population at risk. Limitations may include the quality of data extracted which may not allow studies to be combined in a meta-analysis. This may be overcome by presenting the findings in a descriptive manner. This review will conducted in collaboration with an experienced librarian who helped appraise the search criteria, refine the keywords and MeSH terms and identify appropriate database(s). To the best of our knowledge, no reviews have been published exploring the study question; however, if a review addressing a similar question is published, it will be incorporated in this review and added in a meta-analysis if feasible.

## 17 Implications of results

This systematic review will provide an updated and quantifiable estimate of the risk of stroke in adults with MetS. If it is found that the frequency of stroke is elevated in adults (aged 34–70 years) with stroke. Furthermore, the systematic search will identify where future research is required. For instance, this review may inform a prognostic study which may be useful in understanding the course and factors associated with stroke development.

## Author contributions

LR, ASRB, ALDD, ACF, NPS, PMMD, RMLS, JLO, MN, AD, GBFO, GBZ, SAH, PEOR, AJG, RMP, ACF, PMMD, TMF, NN, SA, CD, PFSG, A.A, AVH, RDL, and FCV conceived the study idea and devised the study methodology. LR, ASRB, ACPC, and ESR participated in the design and coordination of the study. LR was primarily responsible for protocol writing and developed the search strategy. LR and FCV will screen identified literature, conduct data extraction and analyses the review findings. All authors read the drafts, provided comments and agreed on the final version of the manuscript.

**Conceptualization:** Leonardo Roever, Elmiro Santos Resende, Angélica Lemos Debs Diniz, Nilson Penha-Silva, João Lucas O’Connell, Paulo Fernando Silva Gomes, Hugo Ribeiro Zanetti, Anaisa Silva Roerver-Borges, Fernando César Veloso, Thiago Montes Fidale, Antonio Casella-Filho, Paulo Magno Martins Dourado, Antonio Carlos Palandri Chagas, Sadeq Ali-Hasan-Al-Saegh, Paulo Eduardo Ocke Reis, Rogério de Melo Costa Pinto, Gustavo B. F. Oliveira, Alvaro Avezum, Mansueto Neto, André Durães, Rose Mary Ferreira Lisboa da Silva, Antonio José Grande, Celise Denardi, Renato Delascio Lopes, Nitesh Nerlekar, Shahab Alizadeh, Adrian Hernandez Dias, Giuseppe Biondi-Zoccai.

**Formal analysis:** Leonardo Roever.

**Funding acquisition:** Leonardo Roever, Elmiro Santos Resende.

**Methodology:** Leonardo Roever, Elmiro Santos Resende, Angélica Lemos Debs Diniz, Nilson Penha-Silva, João Lucas O’Connell, Paulo Fernando Silva Gomes, Hugo Ribeiro Zanetti, Anaisa Silva Roerver-Borges, Fernando César Veloso, Thiago Montes Fidale, Antonio Casella-Filho, Paulo Magno Martins Dourado, Antonio Carlos Palandri Chagas, Sadeq Ali-Hasan-Al-Saegh, Paulo Eduardo Ocke Reis, Rogério de Melo Costa Pinto, Gustavo B. F. Oliveira, Alvaro Avezum, Mansueto Neto, André Durães, Rose Mary Ferreira Lisboa da Silva, Antonio José Grande, Celise Denardi, Renato Delascio Lopes, Nitesh Nerlekar, Shahab Alizadeh, Adrian Hernandez Dias, Giuseppe Biondi-Zoccai.

**Project administration:** Leonardo Roever.

**Resources:** Leonardo Roever.

**Supervision:** Leonardo Roever.

**Writing – original draft:** Leonardo Roever, Elmiro Santos Resende, Angélica Lemos Debs Diniz, Nilson Penha-Silva, João Lucas O’Connell, Paulo Fernando Silva Gomes, Hugo Ribeiro Zanetti, Anaisa Silva Roerver-Borges, Fernando César Veloso, Thiago Montes Fidale, Antonio Casella-Filho, Paulo Magno Martins Dourado, Antonio Carlos Palandri Chagas, Sadeq Ali-Hasan-Al-Saegh, Paulo Eduardo Ocke Reis, Rogério de Melo Costa Pinto, Gustavo B. F. Oliveira, Alvaro Avezum, Mansueto Neto, André Durães, Rose Mary Ferreira Lisboa da Silva, Antonio José Grande, Celise Denardi, Renato Delascio Lopes, Nitesh Nerlekar, Shahab Alizadeh, Adrian Hernandez Dias, Giuseppe Biondi-Zoccai.

**Writing – review & editing:** Leonardo Roever, Elmiro Santos Resende, Angélica Lemos Debs Diniz, Nilson Penha-Silva, João Lucas O’Connell, Paulo Fernando Silva Gomes, Hugo Ribeiro Zanetti, Anaisa Silva Roerver-Borges, Fernando César Veloso, Thiago Montes Fidale, Antonio Casella-Filho, Paulo Magno Martins Dourado, Antonio Carlos Palandri Chagas, Paulo Eduardo Ocke Reis, Rogério de Melo Costa Pinto, Gustavo B. F. Oliveira, Alvaro Avezum, Mansueto Neto, André Durães, Rose Mary Ferreira Lisboa da Silva, Antonio José Grande, Celise Denardi, Renato Delascio Lopes, Nitesh Nerlekar, Shahab Alizadeh, Adrian Hernandez Dias, Giuseppe Biondi-Zoccai.

## Supplementary Material

Supplemental Digital Content
