# Supplementary material for: Metabolic syndrome and risk of stroke: Protocol for an update systematic review and meta-analysis
Source: Medicine (Baltimore). 2018 Apr 13;97(15):e9862. doi: 10.1097/MD.0000000000009862 (PMC5908602; doi:10.1097/MD.0000000000009862)
Supplement: Supplemental Digital Content [file medi-97-e9862-s001.docx]

**Additional File 1**

**Literature Search Strategy for systematic review of the factors associated Mets and stroke in adults**

**Database 1 (PubMed) search strategy:**

Search: ((((((((stroke/diagnosticand control[MeSH Terms]))) OR metabolic syndrome/stroke[MeSH Terms]))) AND ((((stroke[MeSH Terms]) OR metabolic syndrome [MeSH Terms]) OR stroke[Title/Abstract]) OR OR metabolic syndrome [Title/Abstract])) AND (((((((adults[MeSH Terms]) OR adults[Title/Abstract]) OR adults[Title/Abstract]))

Filters: From 2007/01/01 to 2017/03/31, Humans
